# Unmasking the negative greenhouse effect over the Antarctic Plateau

**DOI:** 10.1038/s41612-018-0031-y

**Published:** 2018-07-11

**Authors:** Sergio A. Sejas, Patrick C. Taylor, Ming Cai

**Affiliations:** 1 NASA Langley Research Center, Climate Science Branch, Hampton, Virginia, USA; 2 Department of Earth, Ocean & Atmospheric Sciences, Florida State University, Tallahassee, FL, USA

## Abstract

A paradoxical negative greenhouse effect has been found over the Antarctic Plateau, indicating that greenhouse gases enhance energy loss to space. Using 13 years of NASA satellite observations, we verify the existence of the negative greenhouse effect and find that the magnitude and sign of the effect varies seasonally and spectrally. A previous explanation attributes this effect solely to stratospheric CO_2_; however, we surprisingly find that the negative greenhouse effect is predominantly caused by tropospheric water vapor. A recently developed principle-based concept is used to provide a complete account of the Antarctic Plateau’s negative greenhouse effect indicating that it is controlled by the vertical variation of temperature and greenhouse gas absorption. Our findings indicate that unique climatological conditions over the Antarctic Plateau—a strong surface-based temperature inversion and scarcity of free tropospheric water vapor—cause the negative greenhouse effect.

## INTRODUCTION

Analogous to a greenhouse, the atmosphere is transparent to incoming solar radiation and opaque to outgoing infrared radiation. This feature allows solar energy to reach the surface while impeding the escape of infrared energy to space, warming Earth’s climate. Put forth by Ekholm in 1901,^1^ the greenhouse analogy ironically fails to explain the main cause of the warming in greenhouses (convective inhibition), but does explain the atmospheric effect, which raises Earth’s global mean surface temperature by ~33 K relative to an “Earth” with no atmosphere. First postulated by Fourier in 1824,^2^ this atmospheric warming effect keeps the Earth from being a desolate ice ball by enabling liquid water to flow freely; thus setting the stage for complex life to develop and evolve.^3^ Aside from variations in solar output, changes in the greenhouse effect (GHE) have driven temperature change throughout Earth’s history and are currently driving anthropogenic climate change through increased carbon dioxide (CO_2_),^4^ whose specific warming qualities were discovered by Tyndall^5^ and implications for global climate first postulated by Arrhenius.^6^

Greenhouse gases such as CO_2_ warm the planet by absorbing the upward longwave (LW) radiation (i.e., infrared radiation) emanating from the surface. Since the atmosphere absorbs the upward LW radiation, it follows that radiation escaping to space does not originate from the ground, but rather from an atmospheric layer at a considerable height above the surface, termed the radiating layer.^7^ The height of the radiating layer is determined by the point where the atmosphere becomes optically transparent. Temperature generally decreases with height above the surface, implying that the radiating layer emits less LW radiation than the surface, reducing energy loss to space.^1,7^ A colder radiating layer relative to the surface implies a greater reduction of energy loss to space and a stronger GHE. The strength of the GHE can thus be quantified by subtracting the outgoing LW radiation (OLR) from the surface LW emission at the same location, with larger positive values indicating a stronger GHE.^8,9^

Before the satellite age in the 1960’s, Earth’s GHE had not been directly measured. Since then, spectral data from satellites has corroborated the hypothesis above, as relative minima are found in the TOA spectrum where greenhouse gases strongly absorb.^10–12^ Unexpectedly, however, an exception occurs over parts of Antarctica for much of the year as relative maxima in the TOA spectrum have been found in spectral bands associated with greenhouse gases,^10,13^ suggestive of a negative GHE. This is a peculiar feature that implies greenhouse gases enhance energy loss to space and cool the climate system, seemingly in contradiction with the long-held view of the GHE.

Applying the radiating layer concept, the negative GHE has been attributed to stratospheric CO_2_ emission, because stratospheric temperatures are typically warmer than the surface over the Antarctic Plateau.^13^ Though it follows a logic similar to the conventional explanation of the positive GHE, this explanation discounts important effects of vertical variations in atmospheric emissivity and temperature. The smaller emissivity of the radiating layer compared to the surface counteracts the effect of the warmer layer. Thus, the temperature difference alone cannot explain the negative GHE. In this study, we present a complete explanation for the peculiar negative GHE and conclude that its existence over the Antarctic Plateau is due predominately to water vapor, not CO_2_.

## RESULTS

### Observed negative GHE

**Fig. 1:**
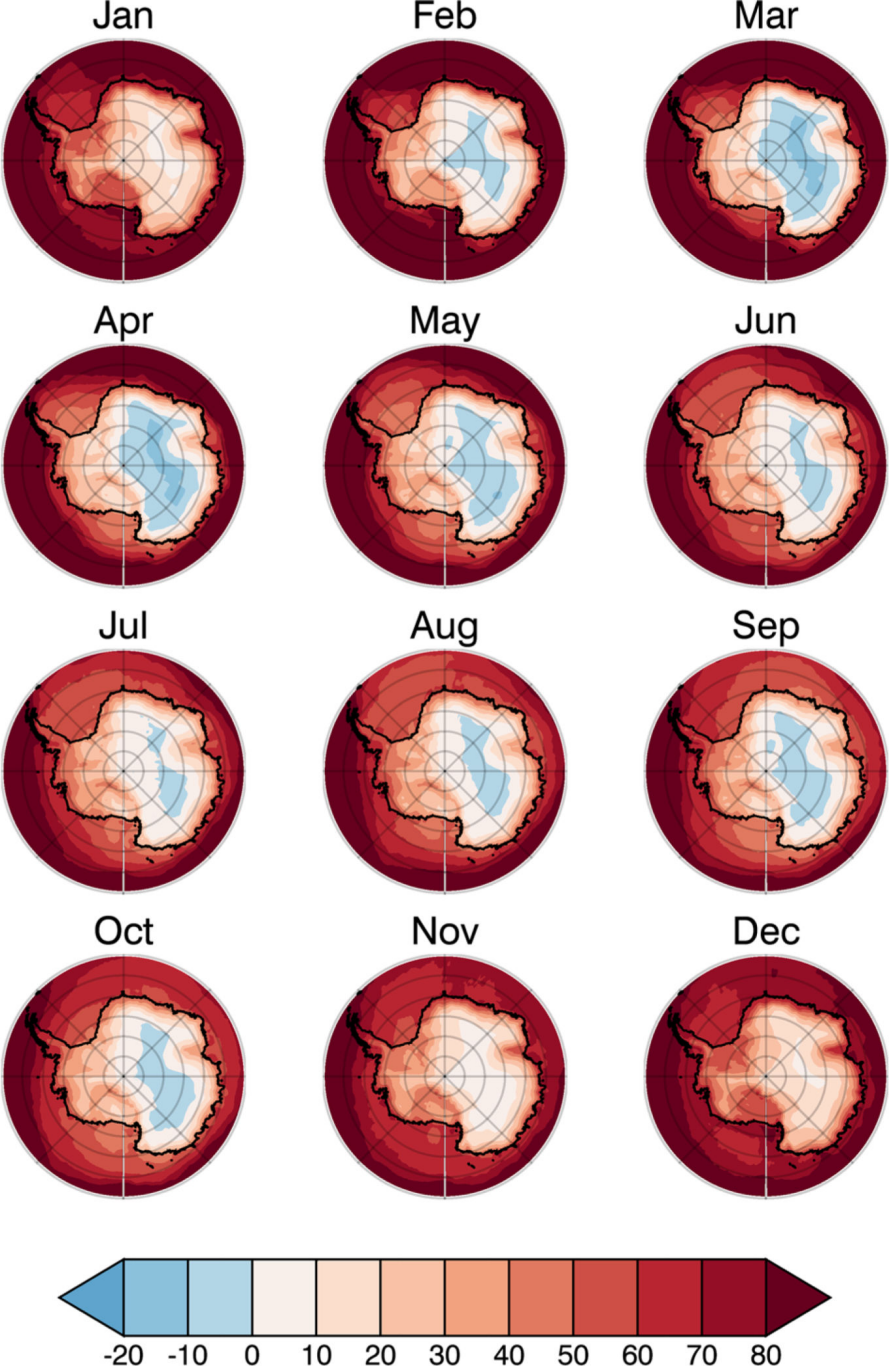
Total GHE strength. The monthly-mean total GHE strength (W*m^−2^) over Antarctica given by AIRS

**Fig. 2:**
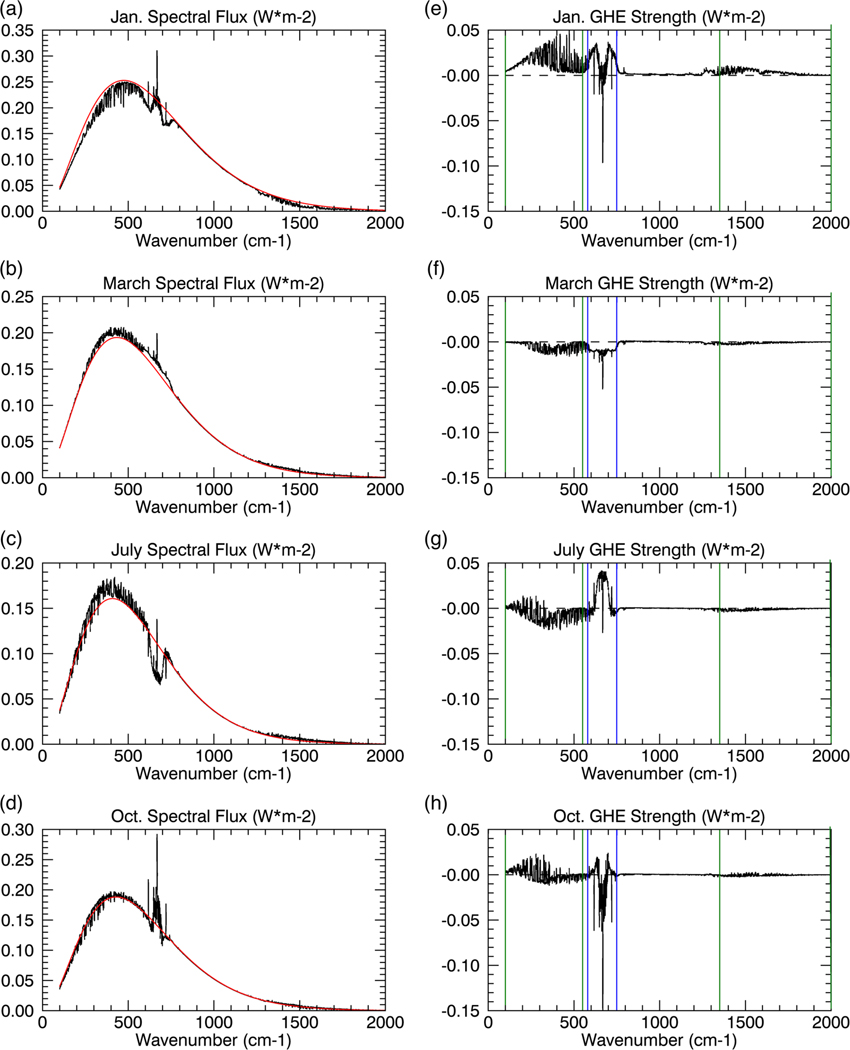
The spectral GHE strength. The calculated spectral upward flux (W*m^−2^) at the surface (red) and TOA (black) for **a** January, **b** March, **c** July, **d** October, and the GHE strength (W*m^−2^) given by the difference between the red and black lines for **e** January, **f** March, **g** July, and **h** October. The vertical green and blue lines delineate the spectral regions in which water vapor and CO_2_ effects dominate, respectively. Calculated for the area-averaged region of the Antarctic Plateau (see Methods section)

Satellite data from NASA’s Atmospheric Infrared Sounder (AIRS)^14^ instruments illustrate the existence of a negative GHE over the Antarctic Plateau during much of the year (blue coloring in Fig. 1). This feature is also found in the NASA’s Clouds and Earth’s Radiant Energy System Energy Balanced and Filled (CERES EBAF)^15,16^ data set and corroborates the negative GHE over the Antarctic Plateau for the same months and with a similar monthly variation (Fig. S1). The negative GHE over the Antarctic Plateau is also corroborated by previous studies with independent data sets.^10,13^ Area-averaged (see Methods section) spectral analyses of the TOA OLR and surface emission reveal that the energy loss to space (Fig. 2; black lines) in spectral regions associated with strong greenhouse gas absorption is greater than surface emission (Fig. 2; red lines); a clear indicator that greenhouse gases enhance the energy loss to space and produce a negative GHE. Unexpectedly, we find the 667 cm^−1^ CO_2_ band (from ~580 to 750 cm^−1^) is not solely responsible for the negative GHE as previously thought.^13^ In addition to the 667 cm^−1^ CO_2_ band, we find water vapor bands (rotational bands below 550 cm^−1^ and vibrational bands above 1350 cm^−1^; Fig. 2) produce a negative GHE.

Seasonally, the negative GHE peaks in both magnitude and areal coverage during March (Fig. 1). In March, the entire 667 cm^−1^ CO_2_ band and all water vapor bands combine to produce a negative GHE (Fig. 2f), with a larger contribution by the water vapor bands to the total negative GHE (Tbl. S1). As austral autumn transitions to winter, the area and magnitude of the negative GHE decreases (Fig. 1). The prolonged winter over the Antarctic Plateau from May to September has a reduced negative GHE due to cancellation between the negative GHE by water vapor and the positive GHE by CO_2_ (Tbl. S1, Fig. 2g). The negative GHE over the Antarctic Plateau during austral winter is thus caused by water vapor alone. During the transition from austral winter to summer in October, there is a reduction of the water vapor negative GHE (Tbl. S1) as the number of water vapor absorption lines with a positive GHE increase (Fig. 2h); CO_2_ therefore becomes the primary cause of the negative GHE observed in October (Fig. 1). During austral summer (i.e., from November to January; Fig. 1), the total negative GHE disappears. The spectral analysis in January, however, reveals that the core of the 667 cm^−1^ CO_2_ band contains a negative GHE that is hidden by the larger positive GHE in the water vapor bands and the wings of the 667 cm^−1^ CO_2_ band. During February, as austral summer transitions to autumn, a similar situation as in October occurs, except the water vapor GHE becomes positive (Tbl. S1), as CO_2_ is responsible for the negative GHE observed in February (Fig. 1). Though CO_2_ clearly contributes to the negative GHE, during the majority of the year (particularly during the prolonged winter) water vapor is dominant cause of the negative GHE. The seasonal picture thus shows that the total negative GHE over the Antarctic Plateau is primarily driven by water vapor.

Figure 2 illustrates that the sign of the GHE varies with wavenumber and season. The sign variation with wavenumber is surprising, since it implies that CO_2_ and water vapor can have opposing effects on the Antarctic Plateau’s seasonal climate. For a given month, the same gas can even have a GHE sign variation depending on wavenumber, illustrated for example by the 667 cm^−1^ CO_2_ band (wings vs. core; Fig. 2e) in January. Whether water vapor and CO_2_ warm or cool the Antarctic climate is determined by the spectral summation of their respective bands.

### Explanation of the negative GHE

**Fig. 3:**
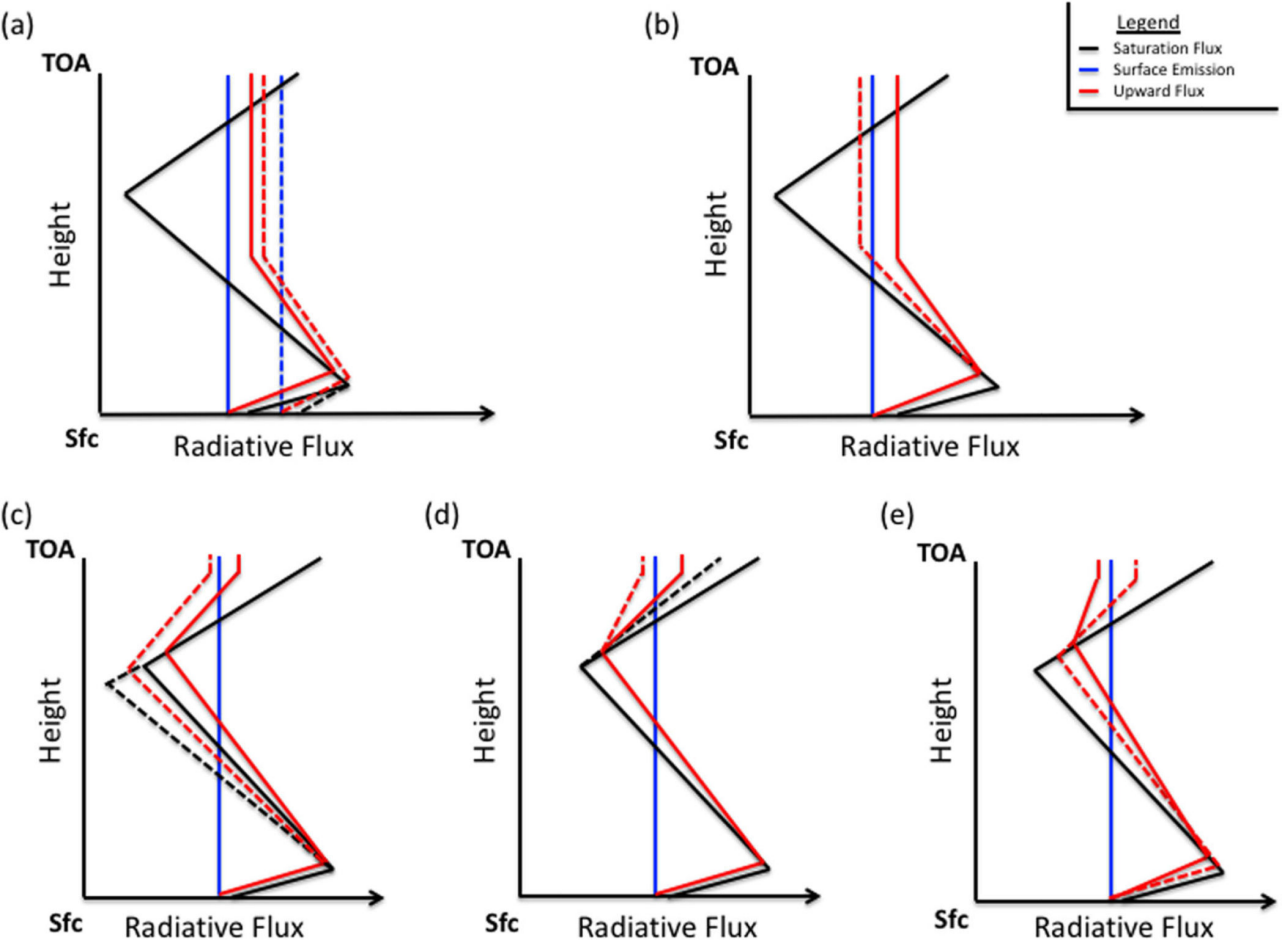
Schematic of different effects on the upward flux. The effects of **a** decreasing the strength of the surface-based temperature inversion layer, **b** increasing the free tropospheric water vapor concentration, **c** increasing the strength of the negative temperature gradient in the free troposphere, **d** decreasing the strength of positive temperature gradient in the stratosphere, and **e** increasing the optical depth in the CO_2_ band on the upward radiative flux (red line) for a temperature profile similar to that over the Antarctic Plateau. The black line is indicative of the blackbody flux, which also serves as a proxy for the vertical temperature profile. The dashed lines illustrate the deviation due to the effects of these modifications from the standard profile (solid). The gap between the surface emission (blue) and upward flux (red) at the TOA is indicative of the strength of the greenhouse effect; the greenhouse effect is negative (positive) when the red line is to the right (left) of the blue line. A validation of the schematic using the LBLRTM is shown in Fig. S2

**Fig. 4:**
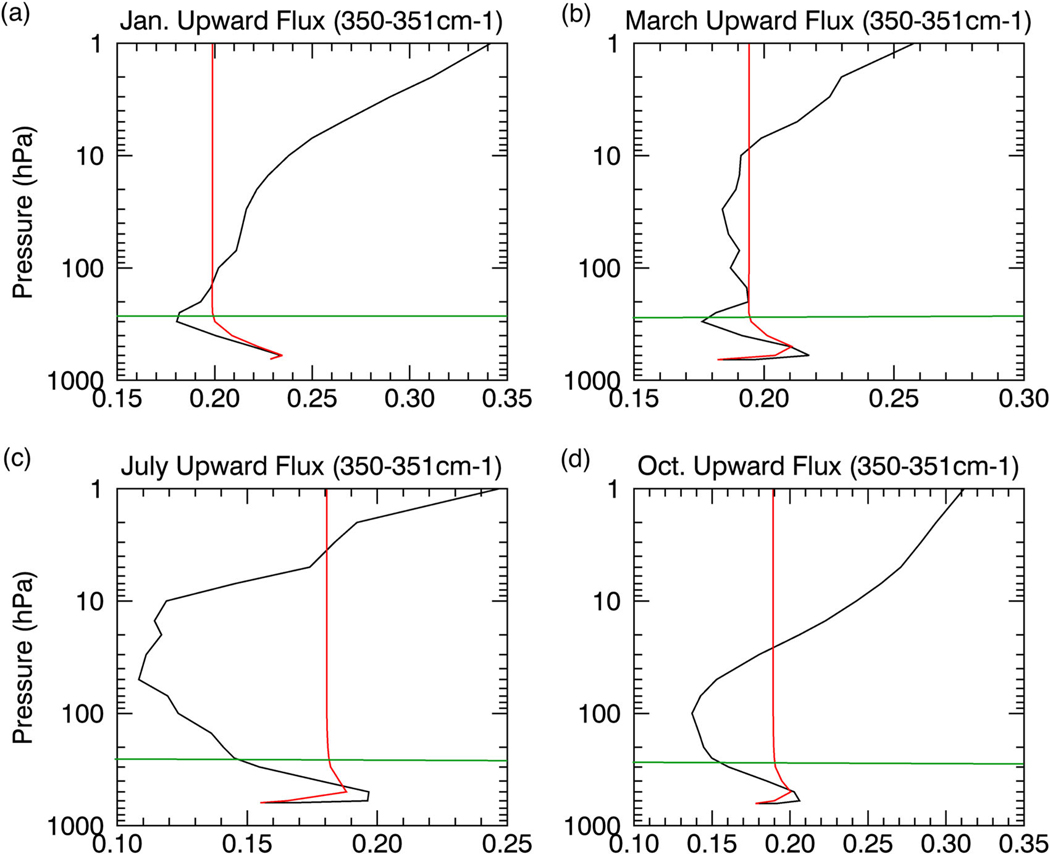
Water vapor effects on the upward flux. The upward flux (W*m^−2^; red) for the 350–351 cm^−1^ band in **a** January, **b** March, **c** July, and **d** October, indicative of the effects of water vapor. The saturation curve (black) is the blackbody flux (W*m^−2^) for the 350–351 cm^−1^ band. The green lines show the approximate height of the radiating layer

**Fig. 5:**
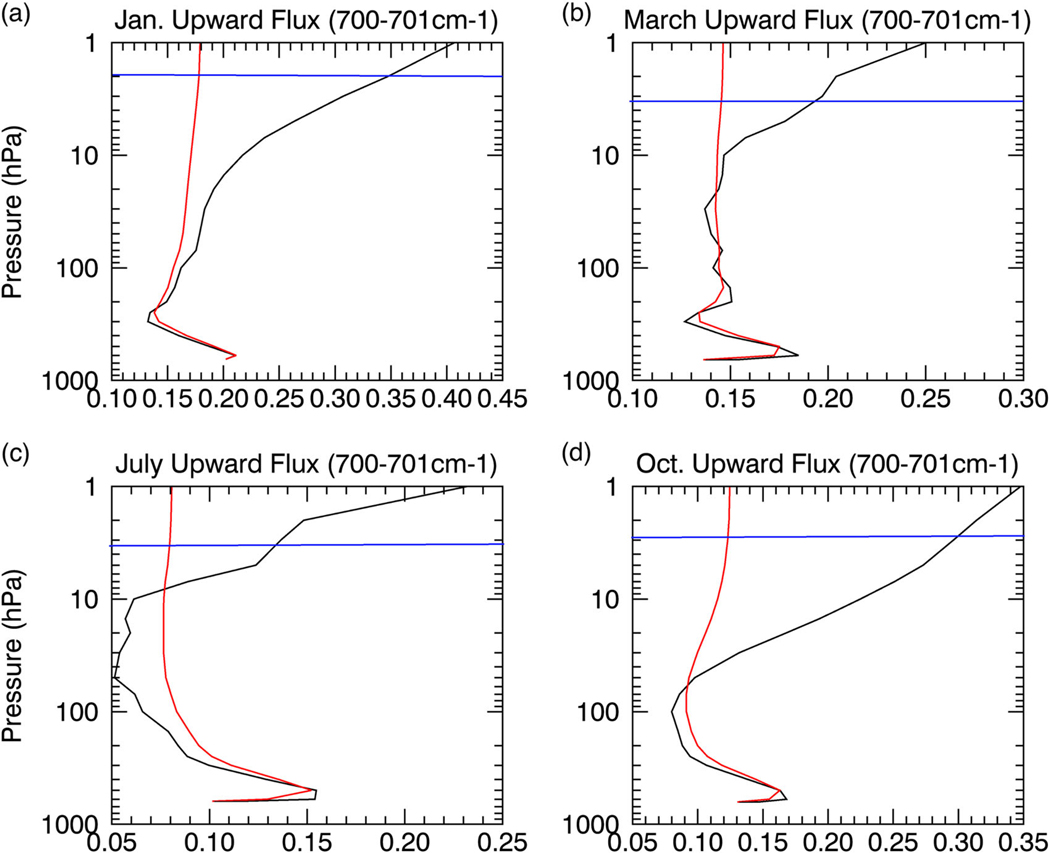
CO_2_ effects on the upward flux in the wings. The upward flux (W*m^−2^; red) for the 700–701 cm^−1^ band in **a** January, **b** March, **c** July, and **d** October, indicative of the effects of CO_2_ toward the wings of the 667 cm^−1^ CO_2_ band. The saturation curve (black) is the blackbody flux (W*m^−2^) for the 700–701 cm^−1^ band. The blue lines show the approximate height of the radiating layer

**Fig. 6:**
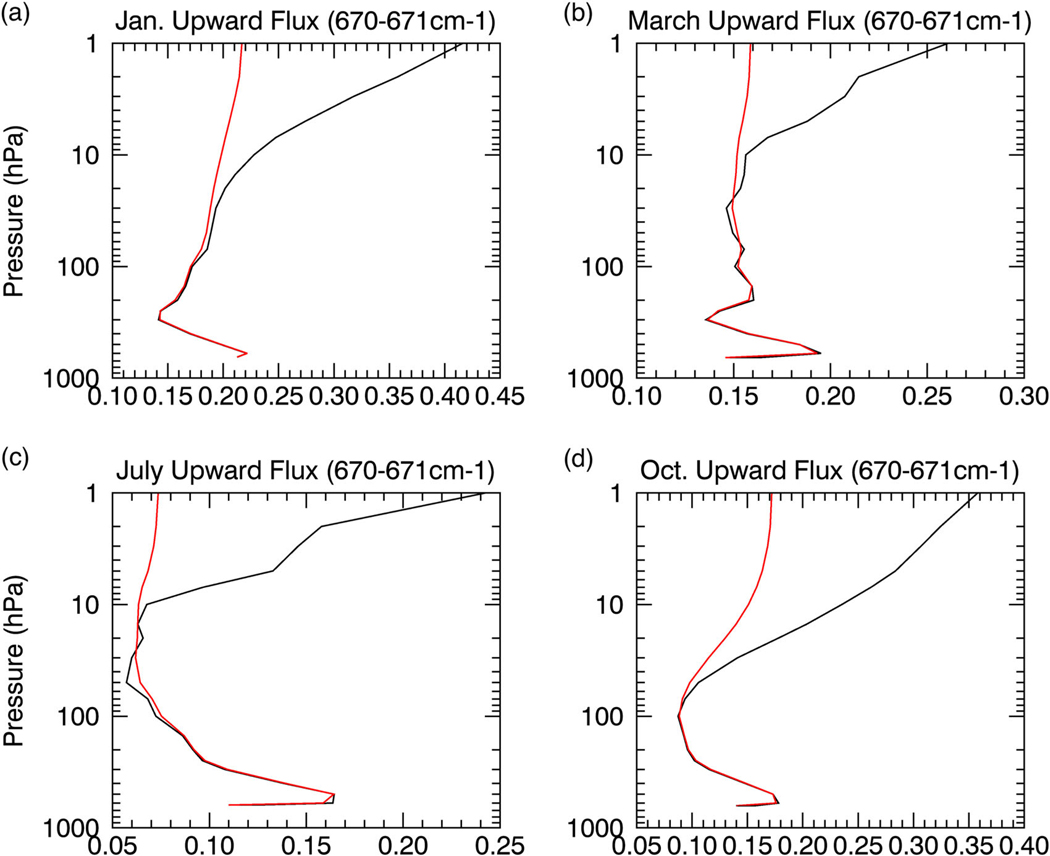
CO_2_ effects on the upward flux in the core. Same as Fig. 5 but for the 670–671 cm^−1^ band, indicative of the effects of CO_2_ in the core of the 667 cm^−1^ CO_2_ band

A recently developed radiative saturation-level concept^17^ summarized in supplementary text, is applied to understand, from a Lagrangian perspective, whether the monochromatic (hereafter dropped but assumed) upward flux emitted by the surface increases, decreases, or remains constant in the presence of absorbers, as it travels from the surface to the TOA. Analogous to the water vapor saturation vapor pressure, the blackbody radiative flux depends only on temperature and defines the radiative saturation point of the upward (and downward) flux; its vertical profile thus establishes a saturation curve that follows the vertical temperature profile. The fundamental principle of the radiative saturation-level concept (schematically illustrated in Fig. 3 and by observational data in Figs. 4–6) is that following the upward flux it always progresses toward the local blackbody flux (i.e., the radiative saturation point) in the presence of absorbers, meaning the upward flux decreases (increases) with height when it is greater (less) than the saturation flux, termed oversaturation (undersaturation).

The difference between the upward and saturation fluxes is mathematically given by the following equation (see supplementary text),

(1) $$F_{v}^{↑}(z)-\pi B_{v}(z)=-\int_{0}^{z}\frac{\partial \pi B_{v}\left(z^{\prime }\right)}{\partial z^{\prime }}T_{v}^{f}(z\prime ,z)dz^{\prime }$$

where $F_{\nu }^{↑}(z)$ and $B_{\nu }(z)$ are the upward flux and Planck function, respectively, at a given height *z* and wavenumber *ν*, and $T_{\nu }^{f}$ is the flux transmittance. As indicated by Eq. (1), the transmittance and vertical blackbody flux gradient (i.e., temperature gradient) determine how close the upward flux is to saturation. For a given temperature gradient, a weaker transmittance results in a smaller difference between the upward flux and blackbody flux, so the absorber amount and strength determines the slope at which the upward flux approaches saturation; greater optical depth yields a stronger approach (Fig. 3b, e and Fig. 5 vs Fig. 6). On the other hand, a stronger vertical temperature gradient increases the gap between the upward and blackbody fluxes, as it makes it harder for the upward flux to “keep up” with the saturation curve as it moves towards saturation. However, if the vertical temperature gradient changes sign in the atmosphere the integral in Eq. (1) indicates there will be offsetting contributions, bringing the upward flux closer to saturation and possibly hitting saturation if the contributions completely offset.

To demonstrate the radiative saturation-level concept, we divide the temperature profile over the Antarctic Plateau into three generalized sections: (1) A lower tropospheric surface-based temperature inversion; (2) a negative temperature gradient in the free troposphere; (3) a positive temperature gradient in the stratosphere. The saturation curve thus increases with height in the lower troposphere and stratosphere but decreases with height in the free troposphere (Figs. 3–6, S2; black line). The upward LW flux (Figs. 3–6, S2 red lines) approaches the saturation curve with the proximity to the saturation curve dependent on the vertical optical depth profile. Its high elevation and polar latitude renders the Antarctic Plateau as the coldest and driest climate on Earth.^18,19^ The extremely low water vapor concentration signifies that the optical depth in water vapor bands will be important only in the lower troposphere, since water vapor concentration and density rapidly decrease above the inversion (Fig. S3). On the other hand, the CO_2_ mixing ratio is uniform but optical depth in the CO_2_ band decreases with height due to the decrease in density (i.e., fewer CO_2_ molecules; Fig. S3). This decrease with height is not overwhelming and the optical depth in the CO_2_ band remains important into the stratosphere.

Due to the near blackbody emission by the surface,^20^ the upward flux begins slightly below the blackbody flux (i.e., undersaturated). The undersaturated nature of the upward flux implies an increase with height (i.e., greater local emission than absorption), as it attempts to keep pace with the increasing saturation curve in the inversion layer (Figs. 3–6); the stronger the inversion (Fig. 3a, S2a and Fig. 4a vs c) and optical depth within the inversion layer (Fig. 3e, S2e and Fig. 5 vs Fig. 6) the greater the increase.

Once the saturation point decreases with height above the inversion, the upward flux crosses the saturation point and becomes oversaturated (>100%; Fig. S4a-d). The oversaturated upward flux will decrease with height (i.e., greater local absorption than emission) tracking the decreasing saturation curve (Figs. 3–6); again, the magnitude of the decrease depends on the optical depth and the rate of temperature decrease (Figs. 3b, c, e, S2b-c, e). Above the inversion, the optical depth for the majority of water vapor absorption lines (below 500 cm^−1^ and above 1350 cm^−1^) rapidly approaches zero, causing the upward flux to decrease slowly. The optical depth in the water vapor bands approaches zero between 200 and 400 hPa (dependent on wavenumber), marking the water vapor radiating layer, above which the upward flux becomes nearly constant to the TOA (Fig. 4, S4). The radiating layer is colder than the surface (Fig. 4), therefore one would expect the TOA flux in the water vapor band to be less than the surface emission. However, as illustrated by the spectral analyses in July and October (Fig. 2c, d), the TOA flux is greater than the surface emission for most water vapor absorption lines demonstrating that the radiating layer concept does not hold.

The combined effects of the strong near-surface temperature inversion and rapidly decreasing water vapor profile above the inversion produce a negative GHE in the majority of water vapor bands for most months (Fig. 2; Tbl. S1), only water vapor absorption lines with strong optical depth, even at low concentrations, will produce a positive GHE. During most months, the weak decrease of the oversaturated upward flux above the surface inversion keeps the upward flux above its surface value enabled by the initial increase of the undersaturated upward flux in the inversion layer. In October, the surface temperature inversion weakens, increasing the number of water vapor absorption lines with a positive GHE, but is still strong enough to produce a negative GHE for the majority of water vapor bands. In summer (i.e., November–January), the surface inversion further weakens causing a smaller initial near-surface increase of the upward flux (Figs. 3a, 4a) that allows the weak upward flux decrease above the surface inversion layer to be strong enough to lower the upward flux below its surface value, eliminating the water vapor negative GHE. During February, as summer transitions to autumn the surface temperature inversion strengthens again, reestablishing a negative GHE for some water vapor absorption lines (not shown), but for a majority of the water vapor bands the inversion is still too weak to produce a negative GHE.

In contrast, the optical depth in the CO_2_ band remains significant for a greater height than for water vapor, so the upward flux decreases below its surface value in the free troposphere (Figs. 3c–e, 5–6, S2c-e). The deeper and stronger the free tropospheric negative temperature gradient the greater the upward flux decrease (Figs. 3c, 5–6, S2c). The only exception is March, since most of the atmosphere is warmer than the surface, keeping the saturation point above its surface value. Thus, in March, the outgoing TOA flux is larger than the surface emission for CO_2_ and all other greenhouse bands (Fig. 2b), indicating a negative GHE.

During all other months, the sign of the GHE in the CO_2_ band also depends on the stratosphere. In the stratosphere, the saturation point once again increases with height eventually exceeding the surface value due to warmer temperatures than the surface. The upward flux therefore once again crosses the saturation point and transitions from decreasing to increasing with height (Figs. 3c–e, 5–6) as it follows the saturation curve. Whether the GHE becomes negative depends on the gap between the upward flux and its surface value at the stratospheric transition point, the local optical depth, and the strength of the positive temperature gradient. The smaller the gap the smaller the upward flux increase needed to surpass the surface value; the stronger the stratospheric positive temperature gradient and optical depth, the greater the upward flux increase with height (Figs. 3d, e, S2d-e). Since optical depth is dependent on wavenumber and the optical depth generally decreases from the center of the 667 cm^−1^ CO_2_ band outwards, the 667 cm^−1^ CO_2_ band core would be more likely to produce a negative GHE than the wings. The optical depth is also dependent on height, so the lower the tropopause the lower the stratospheric transition height and the stronger the optical depth are; therefore, the lower the stratospheric transition from oversaturation to undersaturation occurs the more likely a negative GHE is produced in the 667 cm^−1^ CO_2_ band.

During the prolonged Antarctic winter (i.e., from May to September), the stratospheric upward flux increase is relatively weak since the transition from oversaturation to undersaturation occurs high in the atmosphere, as shown for July in Figs. 5c and 6c, implying a weaker optical depth. The optical depth is too weak at this height for the upward flux increase to surpass its surface value, explaining the positive GHE for the overwhelming majority of the CO_2_ band. During the seasonal change in October, the stratospheric transition from oversaturation to undersaturation occurs at a height much lower than in winter (Fig. 6d), where the optical depth is stronger. The stronger optical depth enhances the stratospheric upward flux increase such that the upward flux surpasses its surface value for the central portion of the 667 cm^−1^ CO_2_ band (from about 640 to 690 cm^−1^). The weaker optical depth in the outer portions of the 667 cm^−1^ CO_2_ band (~580 to 640 cm^−1^ and ~690 to 750 cm ) and higher stratospheric transition height (Fig. 5d) relative to the central portion keep the upward flux increase from surpassing its surface value, thus producing a positive GHE for the outer portions of the 667 cm^−1^ CO_2_ band. In summer, the stratospheric transition occurs at an even lower height than October, as seen during January (Figs. 5a, 6a). However, the large gap between the upward flux value at the stratospheric transition height and its surface value and weaker stratospheric temperature gradient keep the upward flux increase in the stratosphere from surpassing its surface value for most of the 667 cm^−1^ CO_2_ band (Figs. 2e, 5a), but does just surpass its surface value for the very strong optical depth in the central part of the 667 cm^−1^ CO_2_ band (i.e., from ~650 to 680 cm^−1^). During summer the positive GHE for the majority of the 667 cm^−1^ CO_2_ band obscures the negative GHE produced by the central core of the 667 cm^−1^ CO_2_ band. During February, CO_2_ produces a net negative GHE (Tbl. S1) as the stratospheric transition height remains low (as in January) but the gap between the upward flux value at the stratospheric transition height and its surface value is greatly reduced, as the surface begins to cool and the surface temperature inversion strengthens, allowing the stratospheric upward flux increase to surpass its surface value for most of the 667 cm^−1^ CO_2_ band (not shown).

The conventional radiating layer explanation incorrectly attributes the negative GHE in the CO_2_ band solely to the warmer stratospheric temperatures relative to the surface.^13^ Located approximately between 1 and 5 hPa, the CO_2_ band radiating layer is warmer than the surface, but a positive GHE is observed in the CO_2_ band wings (Fig. 5). The radiating layer concept breaks down due to the neglect of the radiating layer emissivity and the variations of vertical emissivity and temperature below it, which dictate the saturation curve and how the upward flux approaches it. Since the saturation curve is dictated by temperature, the more closely the upward flux follows the saturation curve (i.e., greater optical depth), the more likely the radiating layer explanation holds. This explains why the conventional explanation seemingly holds for the CO_2_ band core but breaks down for the CO_2_ band wings.

## DISCUSSION

In general, for a negative GHE to occur temperature must increase with height, driving the maximum saturation value above the surface emission; a condition satisfied over the Antarctic Plateau by warmer stratospheric temperatures relative to the surface and by the surface-based temperature inversion. However, this is a necessary but insufficient condition, as the optical depth determines how efficiently the upward flux moves toward saturation, and a negative temperature gradient above the inversion can cause the upward flux magnitude to decrease below the surface emission. Overall, the entire vertical temperature and optical depth profiles below the TOA determine the magnitude and sign of the GHE. Over the Antarctic Plateau the strong surface-based temperature inversion, persistent for most of the year,^21^ and the scarcity of free tropospheric water vapor above the inversion, are the primary factors that cause the negative GHE.

Over most of the globe, the GHE is positive because stratospheric temperatures warmer than the surface and intense surface inversions are rare, and free tropospheric water vapor is more abundant than over the Antarctic Plateau. Even if the stratosphere were warmer than the surface, producing a negative GHE in the CO_2_ band core, the positive GHE in the water vapor band would obscure this negative GHE, as over the Antarctic Plateau during January (Figs. 1, 2e). Even in the Arctic, where strong, surface-based temperature inversions occur frequently, the greater depth and concentration of water vapor above the inversion drives the upward flux to decrease below the surface value, producing a positive GHE. Our analysis is therefore not contradictory to the well-established and long-held view that greenhouse gases warm the planet. For typical vertical temperature and water vapor profiles the same physics explained by the radiative saturation-level concept dictates that the GHE is positive. Thus, it is the unique climatological conditions over the Antarctic Plateau, which represent an endpoint of terrestrial climate, that cause the negative GHE.

Our analysis reveals that even given the same greenhouse gas mixing ratio, as indicated by the nearly uniform CO_2_ mixing ratio all over the globe, the sign of the GHE strongly depends on the vertical temperature gradient. This dependence on the vertical temperature profile is important, since it implies an increase (decrease) of greenhouse gases does not necessarily enhance (suppress) the GHE, as indicated by the negative radiative forcing produced by increasing the CO_2_ mixing ratio over the Antarctic Plateau.^13,22,23^ While the negative radiative forcing is not responsible for the weak but statistically insignificant surface cooling observed over the Antarctic Plateau,^22,23^ it may partially explain why greenhouse gas increases over Antarctica have not triggered a similar amplified warming response as in the Arctic and provides evidence that observed changes in Antarctica are currently driven by remote connections and internal climate variability.^24^ Moreover, the vertical temperature dependence implies that the strength of the GHE is determined by factors not limited to greenhouse gas mixing ratios. The seasonal temperature profile for example is heavily influenced by the solar insolation,^21^ while the strength of the surface inversion is also dependent on the dynamics.^19^

The newfound understanding of the role of water vapor in producing a negative GHE also has implications to our understanding of past and future climate. A colder Arctic climate in the past (e.g., the ice ages) would imply drier conditions with the potential to produce a negative GHE in water vapor bands, over locations with strong, surface-based temperature inversions (e.g., Greenland); an effect that could have maintained or enhanced the extremely cold climate conditions. As the global climate warms, the redistribution of heat and water vapor by large-scale dynamics could potentially reverse the sign of the GHE over the Antarctic Plateau causing the negative GHE to disappear entirely from the climatological annual cycle. A positive GHE throughout the year over all of Antarctica could potentially make it more similar to the Arctic, which has experienced an amplified warming 2–3 times greater than the global-mean warming over the past 50 years.^25^ Global climate models’ future projections corroborate this speculation, as large warming over the Antarctic continent is projected by the second half of the 21^st^ century.^26,27^ A worrisome prospect as locked up in Antarctica is enough ice to raise sea level by ~73 meters,^28^ melting even a small percentage of that ice would have significant societal impacts.

## METHODS

### Data

The observational monthly data sets are obtained from the AIRS, which has been validated over the Antarctic Plateau region,^29^ and the Clouds and the Earth’s Radiant Energy System (CERES). The AIRS^14^ and CERES^15,16^ data are quality-controlled, averaged, and binned into 1^o^ × 1^o^ grid cells. ‘Climatological’ monthly values were calculated by averaging the 13-year period from 2003–2015, beginning with the first full year of AIRS data. While CERES EBAF provides both OLR and surface LW emission data, AIRS provides OLR data but does not output surface LW emission. AIRS surface skin temperature is used instead to compute the surface LW emission from the Stefan-Boltzmann law, assuming a surface emissivity of 0.99.^20^ The total GHE strength is then estimated by subtracting the OLR $\left(F_{TOA}^{↑}\right)$ from the surface upward LW flux $\left(F_{\text{sfc}}^{†}\right)$,

(2) $$GHE=F_{\text{sfc}}^{†}-F_{\text{TOA}}^{†}$$

### Radiative transfer model

In order to understand the cause of the negative GHE and apply the radiative saturation-level concept, the Line-by-Line Radiative Transfer Model^30^ (LBLRTM) was employed. Since only the LW portion of the spectrum is of interest in this study, wavenumbers from 100 to 2000 cm^−1^ were analyzed using the LBLRTM. Temperature, humidity, ozone, and other greenhouse gas data from AIRS, monthly values averaged over the 13-yr period, were used as input in the LBLRTM to calculate the spectral fluxes over the Antarctic Plateau, which the LBLRTM calculates reasonably well.^18^ Spectral observations from AIRS were also used to validate the LBLRTM calculations. In situ observations indicate AIRS has an approximately vertically uniform cold temperature bias over the Antarctic Plateau, near −3 K on average.^31^ Even with this bias, the vertical structure of the AIRS atmospheric temperature profile agrees well with dropsonde data;^31^ therefore, the influence on the computed negative GHE magnitude is estimated to be less than 10%. The data were area-averaged over the Antarctic Plateau, for months with a negative GHE (Fig. 1) only grid points with a negative GHE were used in the area-average calculation; for months without a negative GHE latitudes between 75°S and 90°S and longitudes between 30.5°E and 120.5°E were used for the area-average calculation. The radiative transfer calculations were done at 24 vertical pressure levels between 1000 and 1 hPa, corresponding to the vertical levels of the AIRS data.

The LBLRTM is able to calculate monochromatic intensities but not monochromatic fluxes. Extremely narrow band fluxes of 1 cm^−1^ width, however, are calculated, which are high resolution enough for the radiative saturation-level concept to approximately hold. The spectral GHE is calculated for every 1 cm^−1^ band by subtracting the upward TOA flux for the given band from the upward surface flux for the same band, similar to the calculation given by Eq. (2). The blackbody flux for every 1 cm^−1^ band is given by

(3) bbflux=πB(T)Δν

where Δν is the band width and *B*(*T*) is the average of the Planck function within that band. The saturation percentage (Figs. S4a-d) is calculated by dividing the upward flux by the blackbody flux and multiplying by 100 at all vertical pressure levels and for all 1 cm^−1^ width bands in the 100–2000 cm^−1^ range.

### Data and code availability

The data that support the findings of this study are available upon request by contacting sergio.sejas@nasa.gov. AIRS data are freely accessible online via https://airs.jpl.nasa.gov/data/get_data. CERES data were obtained from the NASA Langley Research Center CERES ordering tool at http:/ceres.larc.nasa.gov/.Code for the LBLRTM is available for download via http://rtweb.aer.com/.

## Supplementary Material

Supplement
