# Supplementary material for: Unmasking the negative greenhouse effect over the Antarctic Plateau
Source: NPJ Clim Atmos Sci. Author manuscript; Available in PMC 2020 Oct 22. (PMC7580794; doi:10.1038/s41612-018-0031-y)
Supplement: Supplement [file NIHMS1538722-supplement-Supplement.docx]

**Supplementary Information**

**Unmasking the Negative Greenhouse Effect over the Antarctic Plateau**

Sergio A. Sejas^1^, Patrick C. Taylor^1,*^, and Ming Cai^2^

^1^NASA Langley Research Center, Climate Science Branch, Hampton, Virginia, USA

^2^Department of Earth, Ocean & Atmospheric Sciences, Florida State University, Tallahassee, Florida, USA

* To whom correspondence should be addressed. E-mail: patrick.c.taylor@nasa.gov

**Radiative saturation-level concept**

Recently, a new saturation-level concept for radiative transfer was introduced^1^, which aims to physically explain why monochromatic radiative fluxes increase, decrease, or remain constant along their traversal direction. The radiative saturation-level concept classifies the monochromatic flux at a specific point into one of three general saturation levels. The flux is considered saturated if it is equal to the blackbody flux at that specific point. If the flux is less than the blackbody flux it is termed undersaturated. On the other hand, if the flux is greater than the blackbody flux it is termed oversaturated. The blackbody flux is thus the saturation flux, and its vertical profile establishes the saturation curve. For monochromatic fluxes the blackbody flux is dependent only on temperature, so the saturation curve will follow the vertical temperature profile.

When saturated, the flux remains constant along its traversal direction, as the flux emission and absorption are equal, irrespective of the optical depth. This implies that whether the upward or downward flux is being analyzed, the vertical derivative of the flux will be zero when saturated (i.e., $\frac{{dF}_{\nu}^{\uparrow\downarrow}}{dz}=0$). When undersaturated, the flux will increase in the traversal direction, as the flux emission is greater than the absorption; the greater the optical depth the larger the increase. If referring to the upward flux this implies a positive vertical flux gradient (i.e., $\frac{{dF}_{\nu}^{\uparrow}}{dz}>0)$, but a negative vertical flux gradient if referring to the downward flux (i.e., $\frac{{dF}_{\nu}^{\downarrow}}{dz}<0)$. When oversaturated, the flux will decrease in the traversal direction, as the flux absorption is greater than the emission; the greater the optical depth the larger the decrease. If referring to the upward flux this implies a negative vertical flux gradient (i.e., $\frac{{dF}_{\nu}^{\uparrow}}{dz}<0)$, but a positive vertical flux gradient if referring to the downward flux (i.e., $\frac{{dF}_{\nu}^{\downarrow}}{dz}>0)$.

The above discussion implies that as the flux traverses any medium (i.e., the atmosphere) with absorbers (i.e., greenhouse gases) it will always tend towards saturation in its traversal direction; the greater the optical depth, the more readily it will move towards saturation.

This is mathematically indicated by the following equation for the monochromatic upward flux $F_{\nu}^{\uparrow}\left( z \right)$, which is obtained through an integration of Schwarzschild’s equation followed by the use of integration by parts^2^,

$F_{\nu}^{\uparrow}\left( z \right)=\pi B_{\nu}\left( z \right)+\pi\left[ {B_{\nu}^{*}\left( 0 \right)-B}_{\nu}\left( 0 \right) \right]T_{\nu}^{f}\left( 0,z \right)-\int_{0}^{z} \frac{\partial{\pi B}_{\nu}\left( z^{'} \right)}{\partial z^{'}}T_{\nu}^{f}\left( z^{'},z \right)dz'$ (A1),

where *B* is the Planck function, *B^*^* is the Planck function at the skin temperature, and $T_{\nu}^{f}$ is the flux transmittance. The second term on the right-hand side can be considered negligible, which leads to Eq. (1). Eq. (A1) indicates that the closer the transmittance is to zero (i.e., greater optical depth), the closer the upward flux will be to the blackbody flux (i.e., saturation flux). If the magnitude of the flux is already close to the local blackbody flux, an increase in optical depth will have very little effect on the flux magnitude, which is why the blackbody flux is considered the saturation point. However, if there are no absorbers, the optical depth is zero (i.e., transmittance is equal to 1) and the flux will remain constant in the traversal direction, irrespective of its saturation level (e.g., window region).

We should clarify that the radiative saturation-level concept is strictly true only for monochromatic intensities, not fluxes, due to the directional dependency. The concept, however, holds quite well for monochromatic fluxes, particularly if the plane-parallel assumption is used in the radiative calculations. The concept does break down for broadband fluxes though, due to the wavelength dependence of the Planck function and the optical depth. The wavelength dependency of the Planck function means that for every wavelength the saturation curve is different, while for optical depth it implies that the monochromatic fluxes will approach the saturation line at different slopes. Small discrepancies from the conceptual model in Figs. 4-6 arise in part due to the use of a 1 cm^-1^ band in the LBLRTM flux calculation instead of a truly monochromatic calculation. The radiative saturation-level concept should therefore not be applied directly to broadband fluxes, but instead used to explain the spectrum of monochromatic LW fluxes, from which we can extract how the sum of these monochromatic LW fluxes lead to the observed or modeled broadband LW flux.

**References**

1. Sejas, S. A., Cai, M., Liu, G., Taylor, P. C. & Tung, K.-K. A Lagrangian view of longwave radiative fluxes for understanding the direct heating response to a CO2 increase. *J. Geophys. Res. Atmospheres* **121,** 6191–6214 (2016).

2. Goody, R. M. & Yung, Y. L. *Atmospheric Radiation: Theoretical Basis*. (Oxford University Press, 1989).

**Figure Legends**

**Figure S1**. **CERES total GHE strength.** The monthly-mean total GHE strength (W*m^-2^) over Antarctica given by CERES.

**Figure S2.** **Schematic Validation.** Same as Fig. 3 except the schematic is validated using the LBLRTM to illustrate the different effects using area-averaged (see Methods) October atmospheric composition inputs from AIRS. Tbl. S2 indicates how the October atmospheric profiles where perturbed.

**Figure S3**. **Vertical density profiles of greenhouse gases.** The atmospheric density (g/m^3^) of CO_2_ (red) and water vapor (green) molecules in (a) January, (b) March, (c) July, and (d) October.

**Figure S4.** **Radiative saturation-level.** The left column shows the saturation percentage (upward flux divided by the blackbody flux times 100) accompanied by the vertical temperature profile (black line; indicative of the saturation curve) for (a) January, (b) March, (c) July, and (d) October. The right column shows the spectral surface-to-atmosphere upward flux difference (surface emission minus atmospheric upward flux) for (e) January, (f) March, (g) July, and (h) October. Together the two columns explain the upward flux change with height following the radiative saturation-level concept. Calculated for the area-averaged region of the Antarctic Plateau (see Methods).


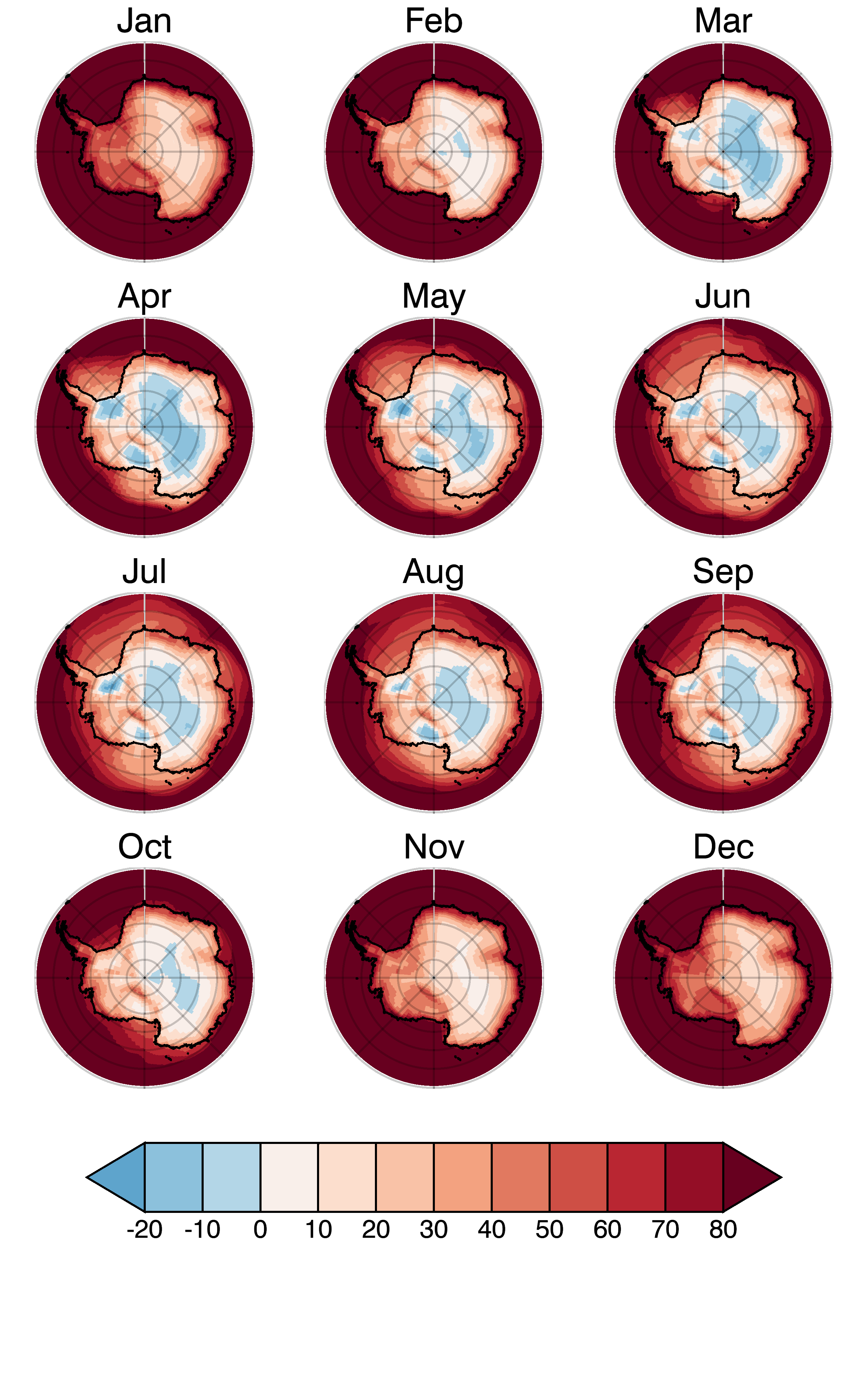


**Fig. S1**. **CERES total GHE strength.** The monthly-mean total GHE strength (W*m^-2^) over Antarctica given by CERES.

**
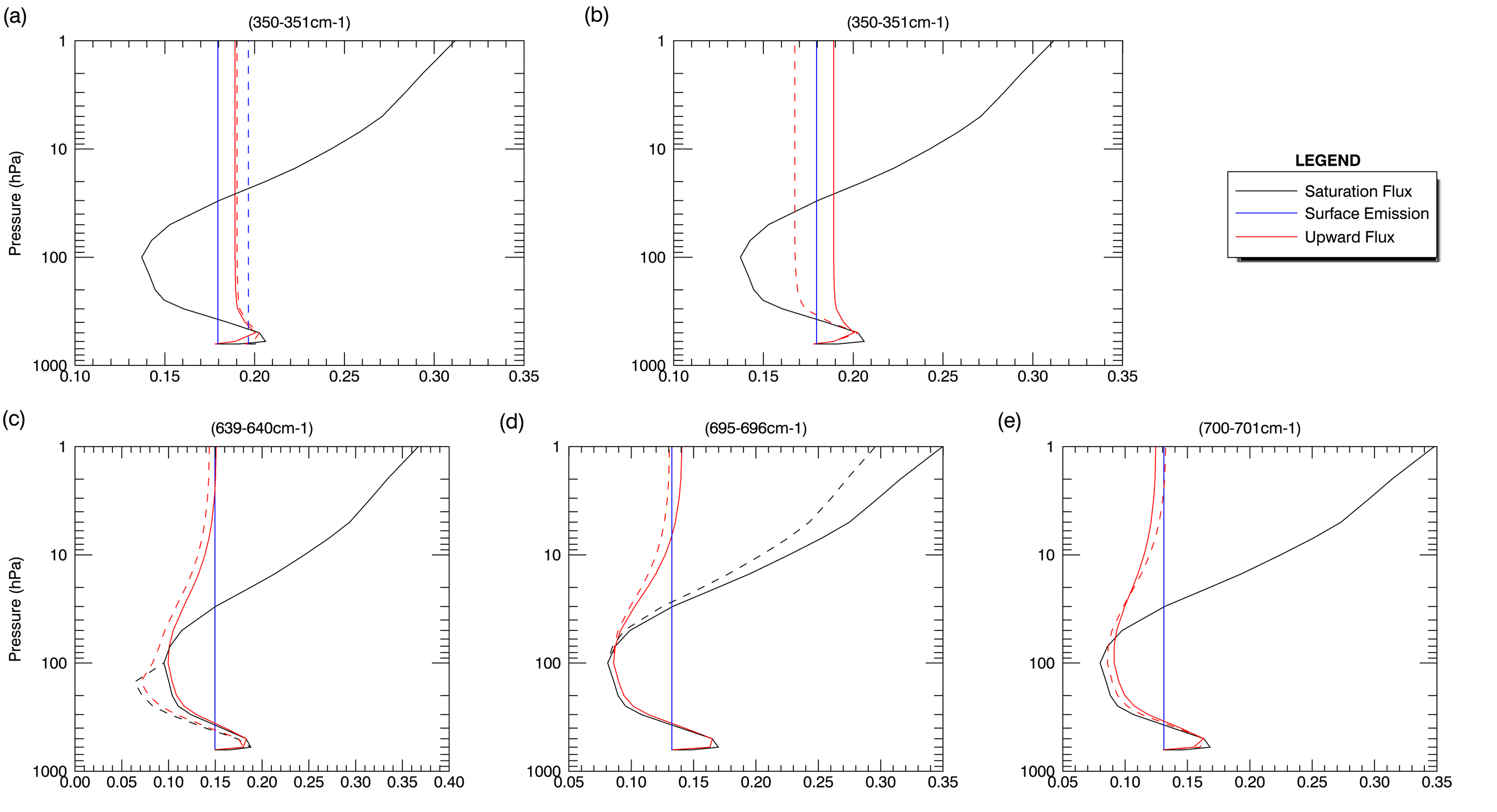
**

**Fig. S2.** **Schematic Validation.** Same as Fig. 3 except the schematic is validated using the LBLRTM to illustrate the different effects using area-averaged (see Methods) October atmospheric composition inputs from AIRS. Tbl. S2 indicates how the October atmospheric profiles where perturbed.


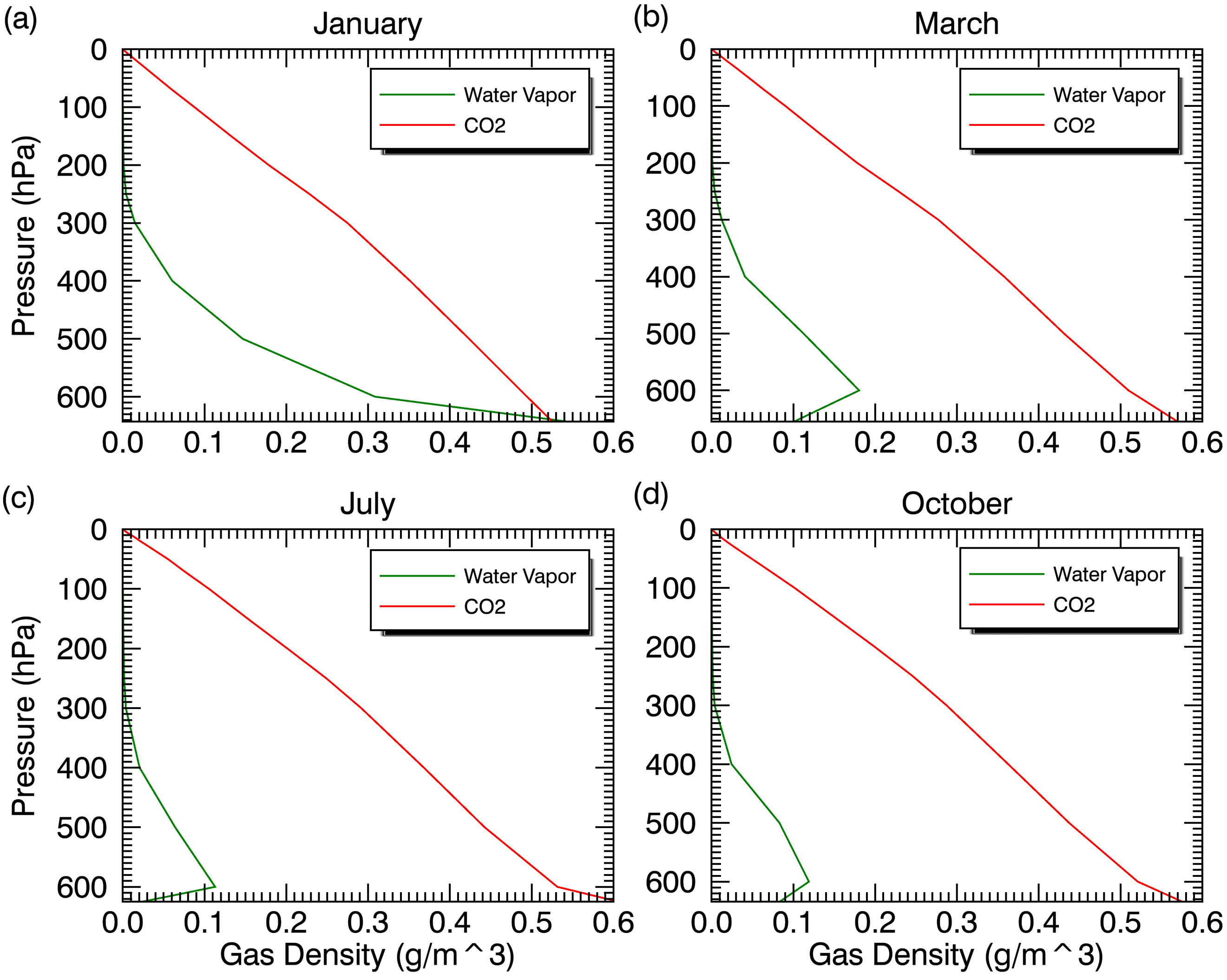


**Fig. S3**. **Vertical density profiles of greenhouse gases.** The atmospheric density (g/m^3^) of CO_2_ (red) and water vapor (green) molecules in (a) January, (b) March, (c) July, and (d) October.


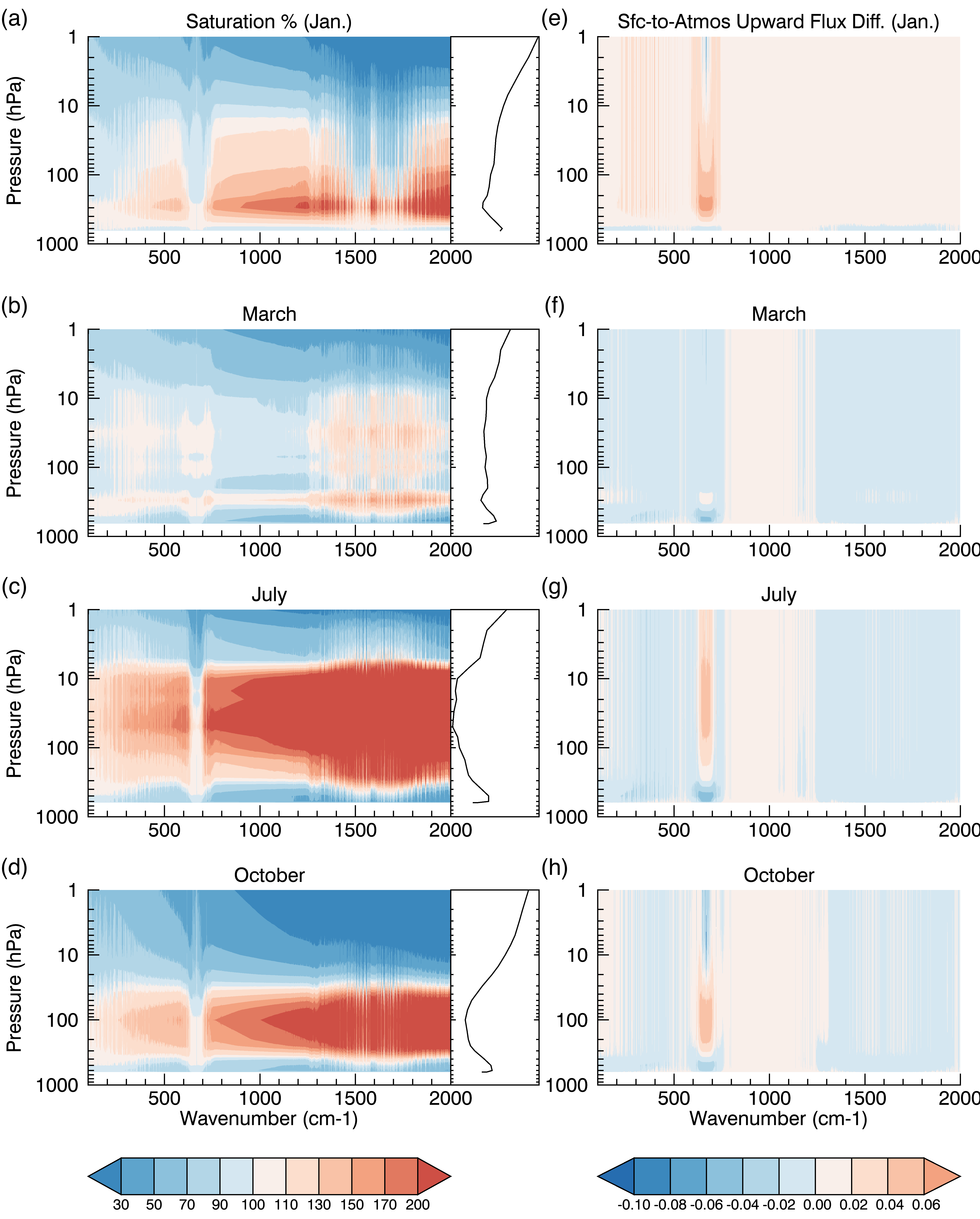


**Fig. S4.** **Radiative saturation-level.** The left column shows the saturation percentage (upward flux divided by the blackbody flux times 100) accompanied by the vertical temperature profile (black line; indicative of the saturation curve) for (a) January, (b) March, (c) July, and (d) October. The right column shows the spectral surface-to-atmosphere upward flux difference (surface emission minus atmospheric upward flux) for (e) January, (f) March, (g) July, and (h) October. Together the two columns explain the upward flux change with height following the radiative saturation-level concept. Calculated for the area-averaged region of the Antarctic Plateau (see Methods).

**Table S1. Monthly net GHE strength in water vapor bands, CO_2_ band, and total LW band.**

| **Month** | **Water Vapor GHE** (W*m^-2^)  **100-550 cm^-1^ and 1350-2000 cm^-1^ Bands** | **CO_2_ GHE** (W*m^-2^)  **580-750 cm^-1^ Band** | **Total GHE** (W*m^-2^)  **100-2000 cm^-1^ Band** |
| --- | --- | --- | --- |
| January | 8.32 | 2.71 | 12.31 |
| February | 0.81 | -1.56 | -0.45 |
| March | -2.87 | -1.85 | -4.83 |
| April | -4.03 | -0.33 | -4.54 |
| May | -3.62 | 1.29 | -2.45 |
| June | -3.68 | 2.09 | -1.69 |
| July | -3.44 | 2.14 | -1.37 |
| August | -3.20 | 1.89 | -1.35 |
| September | -2.54 | 0.84 | -1.69 |
| October | -0.31 | -1.08 | -1.21 |
| November | 5.23 | 1.65 | 6.88 |
| December | 8.37 | 3.38 | 13.15 |

**Table S2. LBLRTM experiments used to validate schematic.**

| **Pressure Level** (hPa) | **October Temperature & H2O Mixing Ratio Profiles** | **Weaker Surface Inversion** | **Greater Free Tropospheric H2O Mixing Ratio** | **Stronger Free Tropospheric Lapse Rate** | **Weaker Stratospheric Temperature Gradient** | **Greater CO_2_ Optical Depth** |
| --- | --- | --- | --- | --- | --- | --- |
| 634.1 | 219.28 K (skin temp) | +8 K | N/A | Same | Same | N/A |
| 633.9 | 224.79 K; 0.084 g/kg | +4 K | Same | Same | Same | 2xCO2 |
| 600 | 231.61 K; 0.132 g/kg | Same | 10xH2O | Same | Same | 2xCO2 |
| 500 | 230.05 K; 0.110 g/kg | Same | 10xH2O | -3 K | Same | 2xCO2 |
| 400 | 221.83 K; 0.039 g/kg | Same | 10xH2O | -6 K | Same | 2xCO2 |
| 300 | 209.96 K; 0.007 g/kg | Same | 10xH2O | -9 K | Same | 2xCO2 |
| 250 | 204.57 K; 0.004 g/kg | Same | 10xH2O | -12 K | Same | 2xCO2 |
| 200 | 201.93 K; 0.002 g/kg | Same | 10xH2O | -15 K | Same | 2xCO2 |
| 150 | 200.43 K; 0.002 g/kg | Same | 10xH2O | -18 K | Same | 2xCO2 |
| 100 | 198.08 K; 0.001 g/kg | Same | 10xH2O | Same | Same | 2xCO2 |
| 70 | 200.92 K; 0.0 g/kg | Same | Same | Same | -1 K | 2xCO2 |
| 50 | 206.11 K; 0.0 g/kg | Same | Same | Same | -2 K | 2xCO2 |
| 30 | 219.48 K; 0.0 g/kg | Same | Same | Same | -3 K | 2xCO2 |
| 20 | 231.55 K; 0.0 g/kg | Same | Same | Same | -4 K | 2xCO2 |
| 15 | 239.16; 0.0 g/kg K | Same | Same | Same | -5 K | 2xCO2 |
| 10 | 247.91 K; 0.0 g/kg | Same | Same | Same | -6 K | 2xCO2 |
| 7 | 254.72 K; 0.0 g/kg | Same | Same | Same | -7 K | 2xCO2 |
| 5 | 260.14 K; 0.0 g/kg | Same | Same | Same | -8 K | 2xCO2 |
| 3 | 265.59 K; 0.0 g/kg | Same | Same | Same | -9 K | 2xCO2 |
| 2 | 269.64 K; 0.0 g/kg | Same | Same | Same | -10 K | 2xCO2 |
| 1.5 | 273.23 K; 0.0 g/kg | Same | Same | Same | -11 K | 2xCO2 |
| 1 | 277.06 K; 0.0 g/kg | Same | Same | Same | -12 K | 2xCO2 |
